# Magnetic Resonance Imaging and Computed Tomography Characteristics of Renal Cell Carcinoma Associated with Xp11.2 Translocation/TFE3 Gene Fusion

**DOI:** 10.1371/journal.pone.0099990

**Published:** 2014-06-13

**Authors:** Wei Wang, Jianhui Ding, Yuan Li, Chaofu Wang, Liangping Zhou, Hui Zhu, Weijun Peng

**Affiliations:** 1 Department of Radiology, Fudan University Shanghai Cancer Center, Shanghai, China; 2 Department of Oncology, Shanghai Medical College, Fudan University, Shanghai, China; 3 Department of Pathology, Fudan University Shanghai Cancer Center, Shanghai, China; University of Washington School of Medicine, United States of America

## Abstract

**Purpose:**

To characterize Xp11.2 translocation renal cell carcinoma (RCC) using magnetic resonance imaging (MRI) and computed tomography (CT).

**Methods:**

This study retrospectively collected the MRI and CT data of twelve patients with Xp11.2 translocation RCC confirmed by pathology. Nine cases underwent dynamic contrast-enhanced MRI (DCE-MRI) and 6 cases underwent CT, of which 3 cases underwent MRI and CT simultaneously. The MRI and CT findings were analyzed in regard to tumor position, size, hemorrhagic, cystic or necrotic components, calcification, tumor density, signal intensity and enhancement features.

**Results:**

The age of the 12 patients ranged from 13 to 46 years (mean age: 23 years). T2WI revealed heterogeneous intensity, hyper-intensity, and slight hypo-intensity in 6 cases, 2 cases, and 1 case, respectively. On DCE-MR images, mild, moderate, and marked rim enhancement of the tumor in the corticomedullary phase (CMP) were observed in 1, 6, and 2 cases, respectively. The tumor parenchyma showed iso-attenuation (n = 4) or slight hyper-attenuation (n = 1) compared to the normal renal cortex on non-contrast CT images. Imaging findings were suggestive of hemorrhage (n = 4) or necrosis (n = 8) in the tumors, and there was evidence of calcification in 8 cases by CT (n = 3) and pathology (n = 8). On dynamic contrast-enhanced CT images, 3 cases and 1 case manifested moderate and strong CMP enhancement, respectively. Nine tumors by MRI and 4 tumors by CT showed prolonged enhancement. Three neoplasms presented at stage I, 2 at stage II, 3 at stage III, and 4 at stage IV according the 2010 AJCC staging criteria.

**Conclusions:**

XP11.2 translocation RCC should be considered when a child or young adult patient presents with a renal tumor with heterogeneous features such as hemorrhage, necrosis, cystic changes, and calcification on CT and MRI and/or is accompanied by metastatic evidence.

## Introduction

Renal cell carcinoma (RCC) associated with Xp11.2 translocation/TFE 3 gene fusion (Xp11.2/TFE 3 RCC or Xp11.2 translocation RCC) is an uncommon subtype of RCC that is now accepted as a distinct entity according to the 2004 World Health Organization renal tumor classification [1]. These tumors are defined by several different translocations in chromosome Xp11.2, resulting in gene fusions in the TFE3 gene. Although some cases have been reported in older patients, Xp11.2/TFE RCC primarily affects children and young adults, and this condition is more common in women than men [1]–[5]. Although Xp11.2 translocation RCCs are less common among adult RCCs than pediatric RCCs, adult Xp11.2 translocation RCCs still out-number pediatric cases [6]. Macroscopically, Xp11.2 translocation RCCs usually manifest tan/yellow and are often accompanied by necrosis and hemorrhage [7]. The associated histopathologic characteristics include clear cell carcinoma with papillary architecture [8]. The most distinctive immunohistochemical feature of this type of tumor is nuclear immunoreactivity for TFE3 protein [8], [9].

Xp11.2 translocation RCC is typically presented as an asymptomatic, painless renal mass and is often identified accidentally by abdominal imaging. Local signs include gross hematuria, flank pain, or a palpable abdominal mass. Initial clinical data suggest an indolent clinical course despite advanced stage. However, recent reports have indicated that adult cases of Xp11.2 translocation RCC have a more aggressive clinical course, and the prognosis of these tumors in adult patients is poor compared to other types of RCC [10]–[12]. The liver, lungs, and retroperitoneal lymph nodes are common sites of metastasis [3].

Radiologic examination may be valuable for the diagnosis of XP11.2 translocation RCC [13]. However, due to the rarity of XP11.2 translocation RCC, only a few case reports focusing on its imaging features are available [11], [13]–[17]. MRI is suggested to be superior to CT due to the increased soft-tissue contrast and multiple imaging weights of MRI, which better reveal the heterogeneous composition (such as hemorrhage or necrosis) of this type of tumor [18]. However, the macroscopic characteristics of XP11.2 translocation RCC are still not well defined by radiological examination due to the small number of imaging reports, and MRI examination is extremely rare. As a result, the noninvasive diagnosis of this type of tumor is difficult, particularly at the early stage.

This study retrospectively collected a relatively large sample of cases of Xp11.2 translocation RCC that had undergone MRI (9 cases) and CT (6 cases) to characterize their imaging features.

## Materials and Methods

### Ethics statement

This retrospective study was approved by the Institutional Review Board of The Fudan Cancer Hospital. All patients provided written informed consent to participate in this study.

### Patients

From January 2009 to June 2013, twelve patients with pathologically proven Xp11.2/TFE RCC were recruited in this study. Eleven of the 12 cases were confirmed by partial or radical nephrectomy and 1 by biopsy (Tables 1–2). One patient died of intraoperative cardiac arrest. None of these patients had received treatment with chemotherapy, surgery, or radiation before the radiological examination. Of the 12 patients, 9 underwent dynamic contrast-enhanced MRI (DCE-MRI) and 6 underwent kidney CT (only 4 were assessed by dynamic contrast-enhanced CT). Three patients underwent MRI and CT simultaneously.

**Table 1:** Clinical and general characteristics of the 12 patients with Xp11.2/TFE RCCs.

No./gender/age/side	Symptoms and signs	*Size(cm)	Regional LN/Capsule/Vessel/M to liver	Stages	Surgical approaches	Follow-up (months)
**1/M/23/R**	Right lumbar pain and gross hematuria	3.5×3.2	+/−/+/−	III	Radical Nx. with para-renal pedicle LN dissection	-
**2/F/46/R**	Right lumbar pain and gross hematuria	3.5×3.5	+/+/+/−	III	Palliative Nx.	-
**3/F/23/R**	Incidentally detected on US	22×20	−/−/−/−	II	Radical Nx.	21
**4/F/15/L**	Low-grade fever and left lumbar pain	20×16	+/+/+/−	IV	Radical Nx.	Dead
**5/F/29/L**	Palpable left abdominal mass	8×7	−/−/−/−	II	Radical Nx.	25
**6/M/13/L**	Interval gross hematuria and left cervical mass	16×17	+/+/+/cervical LN	IV	Radical Nx.	2-whole body metastases
**7/M/33/R**	Right lumbar pain and gross hematuria	7×5	+/−/−/−	III	Radical Nx. with retroperitoneal LN dissection	5
**8/F/25/L**	Incidentally detected on US	3.5×3.5	−/−/−/−	I	Renal adhesiolysis, partial resection, fixation	2
**9/F/36/L**	Gross hematuria	2×2	−/−/−/−	I	Urinary tract resection, Renal adhesiolysis	-
**10/F/40/L**	Left lumbar pain	8×7	+/−/−/liver	IV	Palliative Nx., partial retroperitoneal LN dissection	3-liver, thoracic, lumbar vertebra metastases
**11/M/28/L**	Incidentally detected on US	4×3	−/−/−/−	I	Radical Nx.	10
**12/M/16/R**	Right lumbar pain and gross hematuria	6×4.7	+/+/+/liver	IV	Biopsy	-

*Size: the largest tumor diameter observed on axial scans. LN, lymph node; Nx., nephrectomy. R, Right kidney; L, Left kidney.

**Table 2:** Imaging characteristics of the 12 patients with Xp11.2/TFE RCCs.

No.	Shape/Edge	Location	Hemo/Necrosis/Ca	CT (Enhanced degree/pattern)			MR Signal Intensity		
				PS	CMP	NP	T1WI	T2WI	Enhancement (Degree/Patterns)
**1**	Irreg./WD	Medullary	−/+(P)/+(P)	-	-	-	Iso-	sHypo	Mild Hetero
**2**	Irreg./ID	Medullary	−/−/−	-	-	-	sHyper	Hyper	Moderate Hetero
**3**	Oval/WD	Medullary cortical	+/+/+(P)	-	-	-	Hetero	Hetero	Marked (rim) Hetero
**4**	Irreg./WD	Medullary cortical	+/+/−	-	-	-	Hetero	Hetero	Moderate Hetero
**5**	Irreg./WD	Exophytic	+/+/+(P)	-	-	-	Hetero	Hetero	Moderate Hetero
**6**	Irreg./ID	Medullary cortical	+/+/+(P)	-	-	-	Hetero	Hetero	Markedly (rim) Hetero
**7**	Irreg./ID	Medullary cortical	+(P)/+/+	-	-	Mild Hetero	Hetero	Hetero	Moderate Hetero
**8**	Irreg./ID	Medullary	+(P)/+/+(P)	Iso-	Marked Hetero	Marked Hetero	sHyper	sHyper	Moderate Hetero
**9**	Irreg./WD	Renal pelvis	+(P)/+/+	Iso- Hetero	-	-	sHyper	Hetero	Moderate Hetero
**10**	Irreg./WD	Medullary cortical	+(P)/+(P)/−	sHyper	Moderate sHetero	Moderate Hetero	-	-	-
**11**	Oval/WD	Exophytic	+(P)/+(P)/−	Iso-	Mild sHetero	Mild sHetero	-	-	-
**12**	Oval/ID	Medullary cortical	−/+/+	Hetero	Mild Hetero	Mild Hetero	-	-	-

Note: Irreg., irregular; WD, well-defined; ID, ill-defined; Hemo-, Hemorrhage; Ca, Calcification; P, Pathology; Iso-, isointensity; Hypo-, hypo-intensity; Hyper-, hyper-intensity; Hetero-, heterogeneous; s, slightly; PS, plain (non-contrast) scan; CMP, corticomedullary phase; NP, Nephrographic phase.

### CT and MRI examinations

The parameters of the MRI and CT protocols varied due to the multiple imaging systems used and the retrospective nature of the study.

#### MR acquisition

Seven patients were examined with a 3.0 T whole-body MR system (Signa; GE Medical Systems), and two patients were examined with a 1.5 T twin-speed superconducting MR system (GE Signa with EXCITE II) with 5–8 mm slice thickness and 0.5–2.0 mm gap spacing. MRI sequences included in-phase (IP, TR = 150–230 ms, TE = 1.4–2.4 ms) and opposed-phase (OP, TR = 150–240 ms, TE = 3.2–4.6 ms) T1-weighted axial spoiled gradient echo imaging (SPRG) sequences and fat-saturated T2-weighted axial fast spin echo (FSE) sequences (TR = 3200–4000 ms, TE = 78–92 ms). Intravenous dynamic contrast-enhanced images were obtained in all 9 patients. For the 3T MRI scanner, dynamic contrast-enhanced images were acquired using liver acquisition with a volume acceleration (LAVA) sequence (TR = 2.6–3.2 ms, TE = 1.2–1.5 ms); for the 1.5 T MRI scanner, dynamic contrast-enhanced images were obtained using the T1-weighted FSPGR sequence (TR = 150–180 ms, TE = 1.4–1.7 ms) with fat suppression. The delay time was 20 seconds for CMP, 60 seconds for Nephrographic phase (NP), and 120 seconds for the coronal delayed phase after the intravenous injection of 15 ml of Magnevist (0.1 mmol/kg; Bayer Schering, Pharma AG, Berlin, Germany) at a rate of 2 ml/s.

#### CT acquisition

Six patients were examined with a multi-detector spiral CT machine (Sensation 64, Siemens Medical Systems). Images were obtained at 120 kV and 200 mA with a collimation of 1 mm (n = 4) or 5 mm (n = 5). In 4 of 6 cases, plain (non-contrast) and dynamic contrast images were obtained. Contrast images were acquired in both CMP and NP (n = 4) or Excretory phase (EP) (n = 1). In 1 case, only NP images were acquired, and in 1 patient, only non-contrast CT images were available (patient 9, who also underwent DCE-MRI). The delay time for the contrast-enhanced CT images was 30–35 seconds for CMP, 65–70 seconds for NP and 24 minutes for EP after the intravenous injection of 90 ml iohexol (Omnipaque 300; General Electric) at a rate of 3 ml/s.

### Image interpretation

The imaging characteristics of MRI and CT were retrospectively analyzed by 2 genitourinary radiologists. Both readers were aware that all patients had Xp11.2 translocation RCC but were unaware of the clinical and radiological findings. The images were reviewed on a picture archiving and communication system workstation (GE Aw4.3 workstation).

The evaluated parameters included the tumor position and size, tumor density on unenhanced, CMP, NP or EP CT scans, tumor intensity on T2WI, T1WI or CMP or NP MRI scans, cystic and necrotic changes, hemorrhage, calcification, and metastasis. Readers recorded the size of all renal masses on axial images. The location of the tumor was defined as medullary, cortical, exophytic, or renal pelvis, depending on the relationship of the tumor with the perinephric fat, renal parenchyma, and renal sinus fat. The presence or absence of a tumor boundary was observed and evaluated on the contrast-delayed phase as a clearly defined or poorly defined border. The enhancement pattern of the tumor was classified as homogeneous or heterogeneous, hypo-intense, iso-intense, or hyper-intense. The tumor was classified as a solid or cystic mass depending on the nature of the parenchyma. Intratumoral hemorrhage was considered present if a mass was visually hyper-dense relative to the normal renal parenchyma on non-contrast CT images [19]. Necrosis was defined as the presence of irregular cavitation on contrast-enhanced images [19], [20].

The region of interest (ROI) was defined in the solid portion of the mass to avoid partial volume effects [21]. Each 10-mm ROI was measured 3 times for each image, and the mean value was used. Intratumoral calcification and cystic or necrotic components were excluded from the ROI. The density values of the tumor, normal renal cortex, and medulla were measured on non-contrast and CMP, NP, or EP CT images. Tumor attenuation on unenhanced CT images was classified as ‘mildly high’ if >10 HU, ‘high’ if >30 HU, ‘iso-dense’ if equal to the renal parenchyma, and ‘low’ if <10 HU compared to the normal renal parenchyma [14].

Advanced-stage disease was considered if the images revealed an invasion of the renal vein, a local adenopathy, or distant metastases. Local adenopathy was defined as retroperitoneal nodal enlargement with a short-axis diameter >10 mm [19]. Based on the 2010 AJCC staging criteria, all the renal neoplasms presented at stages I–IV [22].

One primary investigator, Dr. Ding, who was not one of the readers, collected the data and correlated the findings with all available clinical, imaging, and histopathological records, including findings on follow-up imaging and surgery.

## Results

### Clinical and general characteristics of the patients

The patients' medical records were reviewed to evaluate their clinical and general characteristics (Table 1). Follow-up information was also available for 8 cases (Table 1). The age of these patients ranged from 13 to 46 years (mean age: 23 years), and the female to male ratio was 7∶5. Of the 12 patients, 3 patients (25%) were younger than 20 years old, 5 patients (42%) were between 20 and 29 years, 2 patients (17%) were between 30 and 39 years, and 2 patients (17%) were older than 40 years. Most patients (67%) were younger than 30 years of age. Using the 2010 AJCC staging criteria, 3 cases presented at stage I, 2 at stage II, 3 at stage III, and 4 at stage IV.

### CT and MRI features

**Figure 1:**
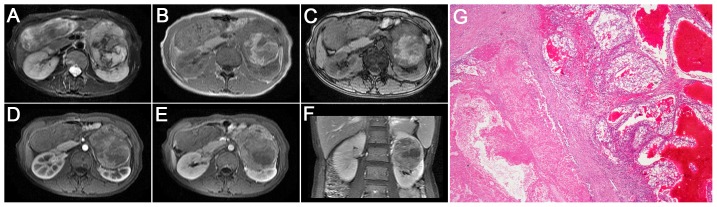
Translocation RCC in a 29-year-old woman (patient 5 in Table 1–2) with a left palpable abdominal mass. **A–C**, Axial T2WI and plain (non-contrast) T1WI (**B**, IP in phase; **C**, OP out of phase) showing a large, well-defined, irregular mass (T2, high-low heterogeneous signal intensity; T1, iso-signal intensity) with a large patchy hemorrhage and necrosis in the center of the mass. **D–F**, CMP and NP gradient-echo MR images showing heterogeneous and prolonged enhancement of the mass. **G**, The tumor cells are polygonal, with voluminous eosinophilic cytoplasm containing a few hyaline nodules and forming a papillary structure (HE 10 & 10).

**Figure 2:**
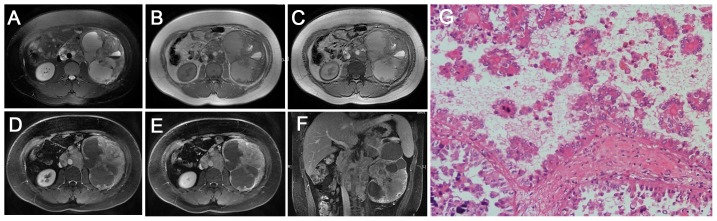
Translocation RCC in a 13-year-old boy (patient 6 in Tables 1–2) with internal gross hematuria and a left cervical mass (biopsy proved metastases). **A–C**, Axial T2WI and plain (non-contrast) T1WI (**B**, IP; **C**, OP) showing a large, ill-defined, irregular mass (T2, high-low mixed signal intensity; T1, iso-high mixed signal intensity; liquid-gas surface) with a large, patchy hemorrhage and necrosis in the center of the left renal mass, without an integrated capsule. Multiple retroperitoneal metastases are detected. **D–E**, CMP and NP gradient-echo MR images showing marked enhancement, predominantly in the periphery of the mass. **F**, Coronal delay phase image showing marked enhancement in the periphery of the mass, invading the left renal pelvic space and vessels. **G**, The tumor cells contain hyaline cytoplasm and show aciniform, papillary and micro-papillary structures (HE 10 & 20).

**Figure 3:**
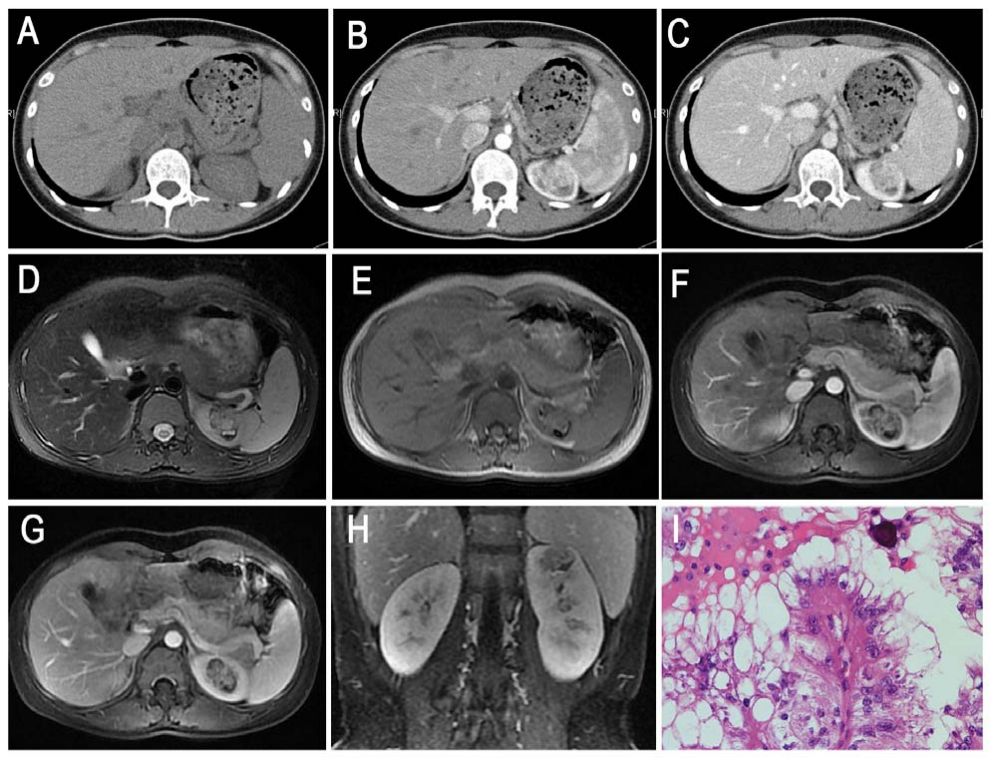
Translocation RCC in a 25-year-old woman (patient 8 in Tables 1, 2, 3) with an incidentally detected left renal mass on US. **A**, Axial unenhanced CT image showing an ill-defined, slightly high attenuation of the mass within the left kidney contour. **B–C**, Axial CMP and NP contrast-enhanced CT images demonstrating heterogeneous and prolonged enhancement of the mass in the left kidney. **D–E**, Axial T2WI and plain (non-contrast) T1WI showing an irregular ill-defined left renal mass with a heterogeneous, slightly high signal intensity, which seemed to break the capsule. Axial CMP (**F**) and NP (**G**) gradient-echo MR images showing heterogeneous moderate enhancement of the left renal mass. **H**, Coronal delay phase image showing delay enhancement of the mass. **I**, The tumor cells contain hyaline cytoplasm, eosinophilic cells with papillary structure and psammoma bodies (HE 10 & 40).

**Figure 4:**
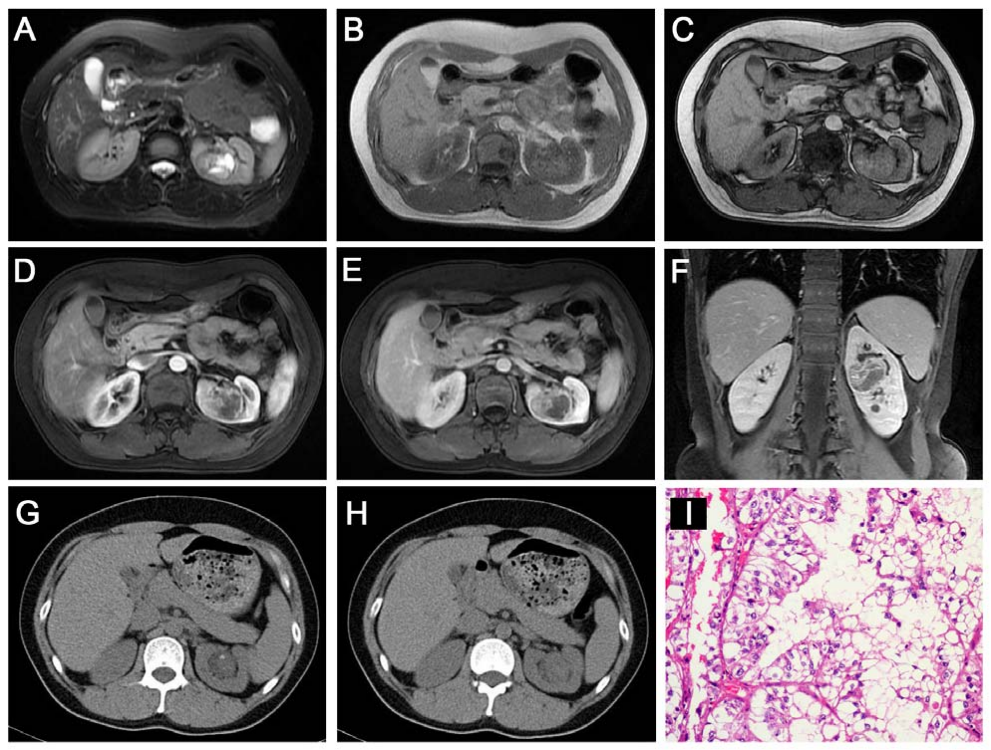
Translocation RCC in a 36-year-old woman (patient 9 in Table 1, 2, 3) with gross hematuria. **A–C**, Axial T2WI and plain (non-contrast) T1WI (**B**, IP in phase; **C**, OP) showing an irregular well-defined mass (T2, iso-high mixed signal intensity; T1, slightly high signal intensity) with a patchy area of cystic necrosis in the left renal pelvis. Axial CMP (**D**) and NP (**E**) gradient-echo MR images showing moderate heterogeneous enhancement in the left renal mass. **F**, Coronal delay phase image showing a delayed-enhancement mass within the contour of the kidney. **G–H**, Unenhanced CT images showing a well-defined, slightly high-density renal mass with mottling calcification. **I**, The tumor cells contain hyaline cytoplasm with a papillary structure (HE 10 & 40).

**Figure 5:**
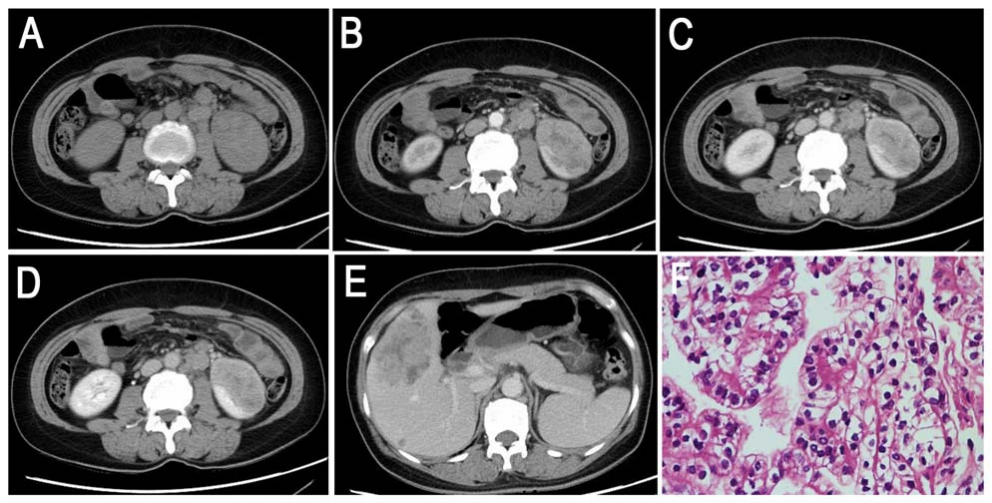
Translocation RCC in a 40-year-old woman (patient 10 in Tables 1, 2, 3) complaining of left flank pain for approximately half a year that became more serious 1 month prior to imaging. **A**, Axial unenhanced CT image showing an ill-defined, slightly higher attenuation soft tissue mass in the left kidney. Retroperitoneal adenopathy is present. **B–D**, Axial CMP, EP and NP contrast-enhanced CT images demonstrating moderate and prolonged enhancement of the mass without an integrated capsule. **E**, Liver metastasis with hemorrhage on EP is observed. **F**, The tumor cells consist of poorly differentiated clear cells (HE 10 & 40).

**Figure 6:**
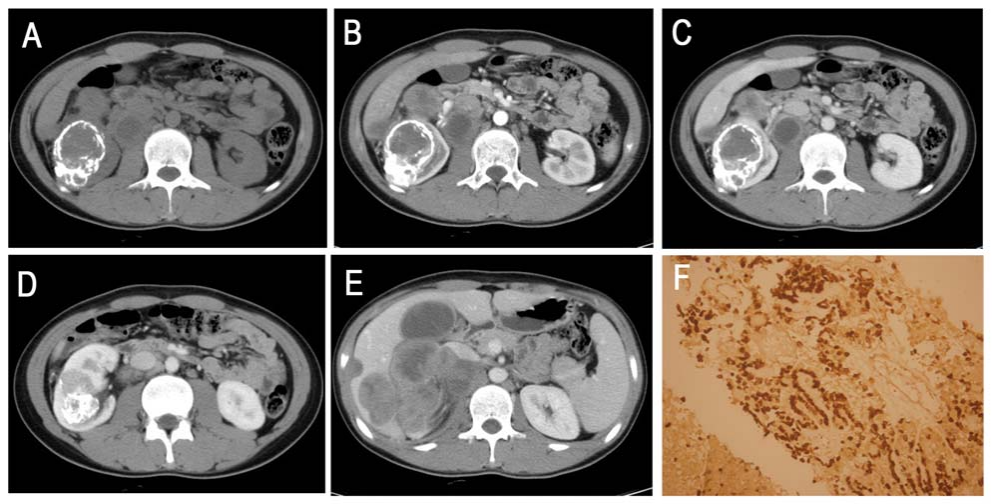
A 16-year-old boy (patient 12 in Tables 1, 2, 3) with XP11.2/TFE 3 confirmed by biopsy. **A**, Axial unenhanced CT image showing an ill-defined, irregular, slightly higher attenuation mass with a bulk of plaque-like calcifications in the right kidney. Retroperitoneal adenopathy is observed. **B–D**, Axial CMP and NP contrast-enhanced CT showing a heterogeneously enhanced mass. **E**, The liver and porta hepatis areas and right retroperitoneal multiple lymphoma metastases are indicated. **F**, Immunohistochemical analysis demonstrated TFE3 nuclear staining (HE 20 & 10).

The imaging characteristics of the 12 patients are summarized in Tables 2 and 3. The representative cases are illustrated in Figures 1, 2, 3, 4, 5, 6.

**Table 3:** HU value of CT attenuation in the 6 patients with Xp11.2/TFE RCCs.

No.	Plain images (Cortex/Medulla/Tumor)	CMP (Cortex/Medulla/Tumor)	NP (Cortex/Medulla/Tumor)	EP (Cortex/Medulla/Tumor)
**7**	-	-	127/88/64	-
**8**	37/22/45	204/67/138	161/240/108	-
**9**	35/25/36	-	-	-
**10**	44/53/59	140/80/82	167/126/101	178//218/100
**11**	36/46/31	119/56/51	138/114/76	-
**12**	40/32/41	162/78/67	164/118/82	-

Note: Hounsfield units (HU); CMP, corticomedullary Phase; NP, nephrographic phase; EP, excretory phase.

The tumor locations included the renal medullary tissue (4 cases, 33%), medullary and cortical tissues (5 cases, 42%), exophytic (2 cases, 17%) and the renal pelvis (1 cases, 8%). In the axial view, 9 lesions were irregular (75%), and the remaining 3 lesions were oval (25%). Seven lesions were well defined (58%), and the remaining 5 were ill defined (42%). All 12 tumors (100%) were surrounded by a fibrous capsule or a false capsule, of which 3 tumors (25%) were presented with infiltrated capsules (Fig. 3D, H). Nine Xp11.2 translocation RCCs (75%) manifested heterogeneous enhancement, and 3 tumors (25%) showed relatively homogeneous enhancement. Eight tumors (67%) were suggested to be accompanied by necrosis and 4 cases (33%) by hemorrhage on plain CT or MRI. There was also evidence of calcification in 3 of 5 CT cases (60%), and 5 of the total 12 cases were confirmed as having calcification by pathology (Fig. 4G, Fig. 6). Retroperitoneal adenopathy (7 cases, 58%) (Fig. 2, 5 and 6) and venous invasion (4 cases, 33%) (Fig. 1, Fig. 2 and Fig. 6) were also detected on MRI or CT.

On T1WI, 4 of the 9 tumors (44%) were slightly hyper-intense or iso-intense (Fig. 3E, Fig. 4B and 4C), and 5 cases (56%) demonstrated an iso-high mixed intensity (Fig. 1–2B, C). On T2WI, 6 of the 9 cases (67%) demonstrated heterogeneous intensity (Fig. 1, 2 and 4), 2 cases (22%) were hyper-intense (Fig. 3D), and 1 case (11%) was slightly hypo-intense. On dynamic contrast-enhanced MRI, 8 of the 9 cases (89%) showed heterogeneous enhancement (Fig. 1, 2 and 4), and 1 case (11%) showed slight inhomogeneous enhancement (Fig. 3). On the CMP, mild and moderate enhancement and marked rim enhancement were observed in 1, 6, and 2 tumors, respectively, and all 9 tumors showed prolonged enhancement.

The imaging characteristics of 6 patients who underwent CT are summarized in Tables 2 and 3. The solid portion of 5 tumors that underwent plain CT showed iso-attenuation (4 cases, 80%) or slight hyper-attenuation (1 case, 20%) compared to the normal renal cortex (Fig. 3–6). On enhanced CT images, 3 of the 4 tumors (75%) showed mild to moderate enhancement, 1 tumor (25%) showed marked enhancement on CMP, and all 4 tumors showed prolonged enhancement (Fig. 3–5). The density was inhomogeneous in 2 cases of plain CT images and in 3 cases of enhanced CT images.

### Histopathologic and immunohistochemical findings

The sizes of the tumors ranged from 2×2 cm to 22×20 cm, with a median size of 10.25 cm. The histopathologic and immunohistochemical features of the tumors from 6 representative cases are shown in Figures 1–6. The tumor cells were polygonal with voluminous eosinophilic cytoplasm and high-grade nuclear features. Eleven tumors (92%) demonstrated cystic portions containing necrosis (11 cases) and hemorrhage (9 cases), 8 tumors (67%) contained calcifications, 5 tumors presented with capsule invasion (42%), 6 tumors (50%) demonstrated retroperitoneal lymph node metastases, and 6 tumors (50%) revealed venous invasion. Immunohistochemical analysis demonstrated TFE3 nuclear staining in all 12 cases, which supported the diagnosis of translocation RCC.

## Discussion

### Clinical characteristics of Xp11.2 translocation RCC

Xp11.2 translocation RCC is a rare subtype that usually affects children and adolescents [23]–[25], with only a few reported adult cases to date [6], [26]. In our study, the age of the patients ranged from 13 to 46 years (67% were younger than 30 years old), which demonstrated a greater average age than that in some reports (median age 13 years) [26], [27] while consistent with that in others [5], [8], [28]. Thus, the presence of RCC in a child or young adult should increase the level of suspicion for Xp11.2 translocation RCC. In our study, the ratio of males to females was 1∶1.4 (5∶7), with a slight female predominance, which is consistent with previous reports [14], [27], although different from the male predominance reported by Dang et al [17]. Little is known about the clinical behavior of these carcinomas [8]. In our study, the symptoms in 75% (9/12) of the patients were nonspecific, and the other 25% (3/12) were detected incidentally. However, most Xp11.2 translocation RCC cases were associated with aggressive clinical course, advanced stage, and relatively poor prognosis in our study. Most of the patients (58%) were at an advanced stage (stage III–IV) although the tumors were small. Adult-onset Xp11.2 translocation RCCs, unlike those with onset during childhood, demonstrate more aggressive clinical course and poorer prognosis, most likely because majority of the adult patients are diagnosed at an advanced clinical stage and do not receive effective systemic therapy [2], [27], [29].

### Imaging characteristics of Xp11.2 translocation RCC

To date, reports on the imaging features of Xp11.2 translocation RCCs are very few. Furthermore, most of them have focused on CT features while only few included MRI [5], [6], [11], [13]–[17]. In this study, we analyzed the imaging manifestations of Xp11.2 translocation RCC using a relatively large sample (9 MRI cases and 6 CT cases); moreover, the combination of MRI and CT may provide additional information regarding this type of RCC. These merits helped to reveal the general imaging features.

#### First, Xp11.2 translocation RCCs mainly involved the renal medullary tissue

Xp11.2 translocation RCCs originate from the renal medulla and are within the contour of the kidney [6]. In our study, most Xp11.2 translocation RCCs were located in the renal medullary tissue or in both the medullary and cortical tissues and within the contour of the kidney, suggesting that these RCCs originated from the renal medulla. The involvement of the pelvic region is occasionally observed, as in our study (1 case) and one early case report [6].

#### Second, the signal intensity on MRI and the density on CT of Xp11.2 translocation RCCs was inhomogeneous, regardless of the tumor size

This may reflect the heterogeneous components within the tumors, such as hemorrhage, necrosis, cystic changes, or calcification [13], [17]. In our study, the CT density of 3 tumors (3/6, 50%) and the MRI signal of 9 tumors (100%) were inhomogeneous. Only a few studies reported that Xp11.2/TFE RCC presents as a homogenously hyper-intense or mildly hyper-intense, dense solid tumor on plain CT images [14]. On plain CT, most of these tumors showed iso-attenuation or slight hyper-attenuation compared to the normal renal cortex. Such hyper-attenuation is thought to be due to hemorrhagic or proteinaceous fluid, densely packed cellular components, or a combination of these factors [13], [16]. MRI provided important information on the tumor parenchyma. Most tumors present heterogeneous mixed signal intensity on T2WI or T1WI because of hemorrhage, necrosis, or cystic changes [17].

#### Third, most of these tumors appeared as a well-defined mass

All 12 cases of XP11.2 translocation RCCs demonstrated complete or near-complete boundaries on MRI/CT (capsule sign), which was best observed during the delayed phase. On T2WI, low-intensity capsule signs were also detected, and ill-defined margins appeared to break the capsule (Fig. 3).

#### Fourth, Xp11.2 translocation RCCs are hypo-vascular tumors and manifest mild/moderate and prolonged enhancement on CT or MRI

On enhanced CT, most tumors in the study were moderately enhanced on CMP and showed prolonged enhancement, and the enhancement of the tumor parenchyma was lower than that of the surrounding normal renal cortex. After the intravenous injection of contrast media on MRI, most tumors (7 of 9) showed heterogeneous enhancement, presenting with cystic-solid changes due to intra-tumoral hemorrhage or necrosis and prolonged enhancement on the solid portion of the tumors. Additionally, the fibrous capsule revealed a gradual rim-like enhancement, which was also consistent with a previous report [13], [17]. Peripheral irregular rims were also present in the tumors around the areas of internal necrosis or hemorrhage, particularly in the larger tumors.

#### Fifth, calcification is an important concomitant sign of Xp11.2 translocation RCCs

In our study, calcifications were detected in 3 of 5 tumors by CT, and 8 of the total 12 cases were confirmed as having calcification by pathology, which was in agreement with previous reports [6], [13]. These calcifications clearly presented as punctate or patchy on CT but were poorly detected by MRI (Fig. 4, Fig. 6). However, calcification is not required to diagnose this subtype, as more than half of the cases did not demonstrate calcification on pathology; one previous study also showed no calcification on unenhanced CT images [14].

#### Finally, cystic and necrotic changes are usually detected in Xp11.2 translocation RCCs

One previous report indicated a unilocular cystic mass with multiple mural nodules on MRI in the right kidney of a 26-year-old pregnant female with Xp11.2/TFE RCC [15]. In our study, most of the tumors demonstrated cystic-solid changes, while none showed such multiple mural nodules. Another early report has shown that tumors contain macroscopic focal fat, which is presumed to be the result of adipose tissue metaplasia [13]. However, no fat component was demonstrated in any of our 12 tumors.

### Differential diagnosis of Xp11.2 translocation RCCs

The differential diagnosis of Xp11.2 translocation RCCs includes clear cell RCC (ccRCC), papillary RCC (pRCC), chromophobe cell RCC (chRCC), collecting duct carcinoma (CDC) and angiomyolipoma (AML) with minimal fat. Most subtypes of RCCs occurs more often in mean ages 50 to 60, and it is rare that patients with Xp11.2 translocation RCCs demonstrate disease at a later age.

ccRCCs have several distinguishing features, including necrosis and hemorrhage, which are commonly associated with lymph node metastasis and renal vein infiltration [23], [27], [28], [30], [31], similar to Xp11.2 translocation RCCs. However, ccRCCs are hyper-vascular tumors and exhibit significantly greater enhancement in the CMP [32]. Stronger enhancement equivalent to the renal cortex is shown only in ccRCCs while not in other subtypes [33]. However, Xp11.2 translocation RCCs present as hypo-vascular tumors with weak prolonged enhancement and clearly differ from ccRCCs.

There are considerable overlaps in morphology between Xp11.2 translocation RCCs and pRCCs, as pRCCs also typically appear hypo-vascular. Homogeneity, small size, regular shape, low-level enhancement, and peripheral location are indicative of a well-differentiated pRCC [30], [34]. pRCCs typically appear as a mass with homogeneous low signal intensity on T2WI, possibly due to cytoplasmic or interstitial histiocytic hemosiderin deposition in the tumor cells [19], [30], [35]–[37]. However, in our study, most XP11.2 translocation RCCs showed heterogeneous signal intensity on T2WI. If pRCCs are poorly differentiated, they can present with heterogeneous attenuation or signal intensity due to necrosis or hemorrhage within the tumor [38], which makes the differential diagnosis with Xp11.2 translocation RCCs difficult.

chRCCs typically localize in the periphery and present as well-defined, large, solid masses without necrosis or calcification; they are commonly diagnosed at an early stage [39]–[41]. In our study, only 1 case (Patient 9 in Table 3) presented with homogenous iso-attenuation and without calcification on plain CT images, which was difficult to distinguish from chRCC. chRCCs also present as hypo-vascular masses, with heterogeneous T2 signal intensity and homogeneous enhancement [18], [40], [41]. Some chRCCs show cystic or necrotic changes in the tumor parenchyma [41], [42], microvascular invasion, local extension, or metastasis, and they are therefore difficult to distinguish from XP11.2 translocation RCCs [43].

CDC is a rare subtype of RCCs that is generally located in the central region of the kidney and typically infiltrates the renal sinus fat. Most of the Xp11.2 translocation RCCs in our study originated in the renal medulla, within the kidney contour. A recent report indicated that the density of Xp11.2 translocation RCCs on unenhanced CT was greater than that of CDC tumors [14].

AMLs with minimal fat are generally hyper-vascular. They typically present with low signal intensity on T2WI and manifest a homogeneously marked and prolonged enhancement pattern on CT [19], [44]. Intra-tumoral calcification can be detected in XP11.2 translocation RCCs, whereas this characteristic is rare in AML.

This study had several limitations. First, because Xp11.2 translocation RCC is an uncommon RCC subtype, selection bias was inevitable. Second, because this study was retrospective, which precluded the careful design of the radiological plans, only routine MRI and CT images were available. Thus, larger samples with advanced MRI techniques, such as diffusion-weighted imaging, perfusion-weighted imaging, and MR spectroscopy, are preferred to improve the imaging diagnostics of this subtype of tumor.

In conclusion, XP11.2 translocation RCC should be considered when a child or young adult patient presents with a renal tumor with the following characteristics: (1) located in the renal medullary tissue; (2) inhomogeneous signal intensity (density) on plain MRI or CT; (3) a well-defined boundary with a fibrous or false capsule; (4) mild/moderate and prolonged inhomogeneous enhancement on CT or MRI; (5) punctate or patchy calcification; (6) necrotic or cystic changes; and (7) signs of advanced stage such as retroperitoneal adenopathy, venous invasion, and distant metastasis. Although this study provides important macroscopic findings for Xp11.2 translocation RCC, the final diagnosis should still be based on microscopic pathology.

## References

[1] Lopez-Beltran A, Scarpelli M, Montironi R, Kirkali Z (2006) 2004 WHO classification of the renal tumors of the adults. Eur Urol 49: 798–805. 16442207 10.1016/j.eururo.2005.11.035

[2] Meyer PN, Clark JI, Flanigan RC, Picken MM (2007) Xp11.2 translocation renal cell carcinoma with very aggressive course in five adults. Am J Clin Pathol 128: 70–79. 17580272 10.1309/LR5G1VMXPY3G0CUK

[3] Winarti NW, Argani P, De Marzo AM, Hicks J, Mulyadi K (2008) Pediatric renal cell carcinoma associated with Xp11.2 translocation/TFE3 gene fusion. Int J Surg Pathol 16: 66–72. 18203790 10.1177/1066896907304994

[4] Altinok G, Kattar MM, Mohamed A, Poulik J, Grignon D, et al (2005) Pediatric renal carcinoma associated with Xp11.2 translocations/TFE3 gene fusions and clinicopathologic associations. Pediatr Dev Pathol 8: 168–180. 15747097 10.1007/s10024-004-9106-3

[5] Salles PG, Soto M Jr (2005) Kidney carcinoma associated with Xp11.2 translocation/TFE3 (ASPL-TFE3) gene fusion. Int Braz J Urol 31: 251–254. 15992428 10.1590/s1677-55382005000300009

[6] Argani P, Olgac S, Tickoo SK, Goldfischer M, Moch H, et al (2007) Xp11 translocation renal cell carcinoma in adults: expanded clinical, pathologic, and genetic spectrum. Am J Surg Pathol 31: 1149–1160. 17667536 10.1097/PAS.0b013e318031ffff

[7] Argani P, Ladanyi M (2003) Recent advances in pediatric renal neoplasia. Adv Anat Pathol 10: 243–260. 12973047 10.1097/00125480-200309000-00001

[8] Argani P, Antonescu CR, Illei PB, Lui MY, Timmons CF, et al (2001) Primary renal neoplasms with the ASPL-TFE3 gene fusion of alveolar soft part sarcoma: a distinctive tumor entity previously included among renal cell carcinomas of children and adolescents. Am J Pathol 159: 179–192. 11438465 10.1016/S0002-9440(10)61684-7 PMC1850400

[9] Argani P, Lal P, Hutchinson B, Lui MY, Reuter VE, et al (2003) Aberrant nuclear immunoreactivity for TFE3 in neoplasms with TFE3 gene fusions: a sensitive and specific immunohistochemical assay. Am J Surg Pathol 27: 750–761. 12766578 10.1097/00000478-200306000-00005

[10] Armah HB, Parwani AV (2008) Renal cell carcinoma in a 33-year-old male with an unusual morphology and an aggressive clinical course: possible Xp11.2 translocation. Pathology 40: 306–308. 18428054 10.1080/00313020701816373

[11] Suzigan S, Drut R, Faria P, Argani P, De Marzo AM, et al (2007) Xp11 translocation carcinoma of the kidney presenting with multilocular cystic renal cell carcinoma-like features. Int J Surg Pathol 15: 199–203. 17478783 10.1177/1066896906295891

[12] Camparo P, Vasiliu V, Molinie V, Couturier J, Dykema KJ, et al (2008) Renal translocation carcinomas: clinicopathologic, immunohistochemical, and gene expression profiling analysis of 31 cases with a review of the literature. Am J Surg Pathol 32: 656–670. 18344867 10.1097/PAS.0b013e3181609914

[13] Koo HJ, Choi HJ, Kim MH, Cho KS (2013) Radiologic-pathologic correlation of renal cell carcinoma associated with Xp11.2 translocation. Acta Radiol 54: 827–834. 23761541 10.1177/0284185113484019

[14] Zhu QQ, Wang ZQ, Zhu WR, Chen WX, Wu JT (2013) The multislice CT findings of renal carcinoma associated with XP11.2 translocation/TFE gene fusion and collecting duct carcinoma. Acta Radiol 54: 355–362. 23446748 10.1258/ar.2012.120255

[15] Armah HB, Parwani AV, Surti U, Bastacky SI (2009) Xp11.2 translocation renal cell carcinoma occurring during pregnancy with a novel translocation involving chromosome 19: a case report with review of the literature. Diagn Pathol 4: 15. 19450277 10.1186/1746-1596-4-15 PMC2690580

[16] Kato H, Kanematsu M, Yokoi S, Miwa K, Horie K, et al (2011) Renal cell carcinoma associated with Xp11.2 translocation/TFE3 gene fusion: radiological findings mimicking papillary subtype. J Magn Reson Imaging 33: 217–220. 21182142 10.1002/jmri.22392

[17] Dang TT, Ziv E, Weinstein S, Meng MV, Wang Z, et al (2012) Computed tomography and magnetic resonance imaging of adult renal cell carcinoma associated with Xp11.2 translocation. J Comput Assist Tomogr 36: 669–674. 23192203 10.1097/RCT.0b013e3182680182

[18] Kondo T, Nakazawa H, Sakai F, Kuwata T, Onitsuka S, et al (2004) Spoke-wheel-like enhancement as an important imaging finding of chromophobe cell renal carcinoma: a retrospective analysis on computed tomography and magnetic resonance imaging studies. Int J Urol 11: 817–824. 15479284 10.1111/j.1442-2042.2004.00907.x

[19] Silverman SG, Mortele KJ, Tuncali K, Jinzaki M, Cibas ES (2007) Hyperattenuating renal masses: etiologies, pathogenesis, and imaging evaluation. Radiographics 27: 1131–1143. 17620471 10.1148/rg.274065147

[20] Studer UE, Scherz S, Scheidegger J, Kraft R, Sonntag R, et al (1990) Enlargement of regional lymph nodes in renal cell carcinoma is often not due to metastases. J Urol 144: 243–245. 2374186 10.1016/s0022-5347(17)39422-3

[21] Sauk SC, Hsu MS, Margolis DJ, Lu DS, Rao NP, et al (2011) Clear cell renal cell carcinoma: multiphasic multidetector CT imaging features help predict genetic karyotypes. Radiology 261: 854–862. 22025734 10.1148/radiol.11101508

[22] Edge SB, Compton CC (2010) The American Joint Committee on Cancer: the 7th edition of the AJCC cancer staging manual and the future of TNM. Ann Surg Oncol 17: 1471–1474. 20180029 10.1245/s10434-010-0985-4

[23] Argani P, Ladanyi M (2006) The evolving story of renal translocation carcinomas. Am J Clin Pathol 126: 332–334. 16880145 10.1309/EAEJTJGD5J4J3B4F

[24] Argani P, Ladanyi M (2005) Translocation carcinomas of the kidney. Clin Lab Med 25: 363–378. 15848741 10.1016/j.cll.2005.01.008

[25] Argani P, Lae M, Hutchinson B, Reuter VE, Collins MH, et al (2005) Renal carcinomas with the t(6;11)(p21;q12): clinicopathologic features and demonstration of the specific alpha-TFEB gene fusion by immunohistochemistry, RT-PCR, and DNA PCR. Am J Surg Pathol 29: 230–240. 15644781 10.1097/01.pas.0000146007.54092.37

[26] Wu A, Kunju LP, Cheng L, Shah RB (2008) Renal cell carcinoma in children and young adults: analysis of clinicopathological, immunohistochemical and molecular characteristics with an emphasis on the spectrum of Xp11.2 translocation-associated and unusual clear cell subtypes. Histopathology 53: 533–544. 18983462 10.1111/j.1365-2559.2008.03151.x

[27] Rao Q, Chen JY, Wang JD, Ma HH, Zhou HB, et al (2011) Renal cell carcinoma in children and young adults: clinicopathological, immunohistochemical, and VHL gene analysis of 46 cases with follow-up. Int J Surg Pathol 19: 170–179. 20034980 10.1177/1066896909354337

[28] Argani P, Lui MY, Couturier J, Bouvier R, Fournet JC, et al (2003) A novel CLTC-TFE3 gene fusion in pediatric renal adenocarcinoma with t(X;17)(p11.2;q23). Oncogene 22: 5374–5378. 12917640 10.1038/sj.onc.1206686

[29] Aoyagi T, Shinohara N, Kubota-Chikai K, Kuroda N, Nonomura K (2011) Long-term survival in a patient with node-positive adult-onset Xp11.2 translocation renal cell carcinoma. Urol Int 86: 487–490. 21335945 10.1159/000323866

[30] Pedrosa I, Chou MT, Ngo L, R HB, Genega EM, et al (2008) MR classification of renal masses with pathologic correlation. Eur Radiol 18: 365–375. 17899106 10.1007/s00330-007-0757-0

[31] Eren S, Karaman A, Okur A (2006) The superior vena cava syndrome caused by malignant disease. Imaging with multi-detector row CT. Eur J Radiol 59: 93–103. 16476534 10.1016/j.ejrad.2006.01.003

[32] Young JR, Margolis D, Sauk S, Pantuck AJ, Sayre J, et al (2013) Clear cell renal cell carcinoma: discrimination from other renal cell carcinoma subtypes and oncocytoma at multiphasic multidetector CT. Radiology 267: 444–453. 23382290 10.1148/radiol.13112617

[33] Fujimoto H, Wakao F, Moriyama N, Tobisu K, Sakamoto M, et al (1999) Alveolar architecture of clear cell renal carcinomas (< or = 5.0 cm) show high attenuation on dynamic CT scanning. Jpn J Clin Oncol 29: 198–203. 10340043 10.1093/jjco/29.4.198

[34] Herts BR, Coll DM, Novick AC, Obuchowski N, Linnell G, et al (2002) Enhancement characteristics of papillary renal neoplasms revealed on triphasic helical CT of the kidneys. AJR Am J Roentgenol 178: 367–372. 11804895 10.2214/ajr.178.2.1780367

[35] Yoshimitsu K, Irie H, Tajima T, Nishie A, Asayama Y, et al (2004) MR imaging of renal cell carcinoma: its role in determining cell type. Radiat Med 22: 371–376. 15648450

[36] Sasiwimonphan K, Takahashi N, Leibovich BC, Carter RE, Atwell TD, et al (2012) Small (<4 cm) renal mass: differentiation of angiomyolipoma without visible fat from renal cell carcinoma utilizing MR imaging. Radiology 263: 160–168. 22344404 10.1148/radiol.12111205

[37] Sussman SK, Glickstein MF, Krzymowski GA (1990) Hypointense renal cell carcinoma: MR imaging with pathologic correlation. Radiology 177: 495–497. 2217790 10.1148/radiology.177.2.2217790

[38] Delahunt B, Eble JN, McCredie MR, Bethwaite PB, Stewart JH, et al (2001) Morphologic typing of papillary renal cell carcinoma: comparison of growth kinetics and patient survival in 66 cases. Hum Pathol 32: 590–595. 11431713 10.1053/hupa.2001.24984

[39] Amin MB, Paner GP, Alvarado-Cabrero I, Young AN, Stricker HJ, et al (2008) Chromophobe renal cell carcinoma: histomorphologic characteristics and evaluation of conventional pathologic prognostic parameters in 145 cases. Am J Surg Pathol 32: 1822–1834. 18813125 10.1097/PAS.0b013e3181831e68

[40] Sasaguri K, Irie H, Kamochi N, Nakazono T, Yamaguchi K, et al (2010) Magnetic resonance imaging of large chromophobe renal cell carcinomas. Jpn J Radiol 28: 453–459. 20661696 10.1007/s11604-010-0450-0

[41] Rini BI, Wilding G, Hudes G, Stadler WM, Kim S, et al (2009) Phase II study of axitinib in sorafenib-refractory metastatic renal cell carcinoma. J Clin Oncol 27: 4462–4468. 19652060 10.1200/JCO.2008.21.7034

[42] Pedrosa I, Sun MR, Spencer M, Genega EM, Olumi AF, et al (2008) MR imaging of renal masses: correlation with findings at surgery and pathologic analysis. Radiographics 28: 985–1003. 18635625 10.1148/rg.284065018

[43] Vera-Badillo FE, Conde E, Duran I (2012) Chromophobe renal cell carcinoma: a review of an uncommon entity. Int J Urol 19: 894–900. 22715810 10.1111/j.1442-2042.2012.03079.x

[44] Kim JK, Park SY, Shon JH, Cho KS (2004) Angiomyolipoma with minimal fat: differentiation from renal cell carcinoma at biphasic helical CT. Radiology 230: 677–684. 14990834 10.1148/radiol.2303030003

